# Bystanders, parcelling, and an absence of trust in the grooming interactions of wild male chimpanzees

**DOI:** 10.1038/srep20634

**Published:** 2016-02-09

**Authors:** Stefano S. K. Kaburu, Nicholas E. Newton-Fisher

**Affiliations:** 1 Department of Population Health and Reproduction, School of Veterinary Medicine, University of California Davis, Davis, CA, USA; 2 Living Primates Research Group, School of Anthropology and Conservation, Marlowe Building, University of Kent, Canterbury, Kent CT2 7NR, UK

## Abstract

The evolution of cooperation remains a central issue in socio-biology with the fundamental problem of how individuals minimize the risks of being short-changed (‘cheated’) should their behavioural investment in another not be returned. Economic decisions that individuals make during interactions may depend upon the presence of potential partners nearby, which offers co operators a temptation to defect from the current partner. The parcelling model posits that donors subdivide services into parcels to force cooperation, and that this is contingent on opportunities for defection; that is, the presence of bystanders. Here we test this model and the effect of bystander presence using grooming interactions of wild chimpanzees. We found that with more bystanders, initiators gave less grooming at the beginning of the bout and were more likely to abandon a grooming bout, while bouts were less likely to be reciprocated. We also found that the groomer’s initial investment was not higher among frequent groomers or stronger reciprocators, suggesting that contrary to current assumptions, grooming decisions are not based on trust, or bonds, within dyads. Our work highlights the importance of considering immediate social context and the influence of bystanders for understanding the evolution of the behavioural strategies that produce cooperation.

In group-living species, many social interactions occur in the presence of others. The influence of such bystanders has attracted increasing attention, both in their role as an audience that influences performance (audience effect: e.g. *Gallus gallus domesticus*^1^, *Pan troglodytes*^2^), and as eavesdroppers who acquire information that they can later exploit^3^ (e.g. *Astatotilapia burtoni*^4^, *Procambarus clarkij *5). This interest has led to investigation into the importance of bystanders as a selective influence on the evolution of cooperative behaviour; eavesdroppers may benefit by identifying individuals with whom they can cooperate in future^3^. The focus on the information extracted by bystanders^4^,^5^, and whether actors attempt to manipulate this information (e.g. *Betta splendens*^6^, *Pongo* spp.^7^), however overshadows a more direct role for bystanders in the evolution of cooperation through widening the range of options open to actors by offering alternative partners with which to cooperate.

Understanding the evolution of cooperation in social animals remains a central issue in socio-biology^8^,^9^, with the fundamental problem of how individuals minimize the risks of being short-changed (‘cheated’) should their behavioural investment in another not be returned. Economic decisions that individuals make when interacting with group members may well depend upon the presence of potential partners nearby, and it is therefore vital to determine the extent to which individuals respond to bystander presence^10^,^11^. The importance of bystanders is highlighted in two particular theoretical approaches, Noë and colleagues’^10^,^11^ biological markets theory (BMT) and Connor’s parcelling model^12^,^13^ (see also Friedman & Hammerstein^14^) for the evolution of cooperative exchanges.

From a purely dyadic perspective, Connor’s parcelling seems little different to a tit-for-tat strategy^15^: one individual provides a small level of cooperative investment, and continues to do so as long as its partner does likewise^12^. However, central to Connor’s model is the concept that the existence of bystanders generates a temptation to defect from the current partner, and that this drives the parcelling (subdividing) of a social interaction such that the partner is forced into cooperating in order to extract significant benefit from the interaction, while minimising the cost to the actor should the partner defect (i.e. refuse to cooperate or select another partner)^12^,^13^. When individuals have easy opportunities to find alternative partners, such that the costs of searching are low, defection − the lack of reciprocation − is more likely to occur and the donor should give smaller parcels in order to limit its losses^12^,^13^.

Superficially similar to parcelling is Roberts & Sherratt’s raise-the-stakes (RTS)^16^ model, which proposes that cooperative acts should be delivered through a pattern of variable investment. However, unlike parcelling, RTS is a purely dyadic model that does not consider the potential influence of bystanders. RTS actors are concerned solely with their investment, and whether or not it is matched; the presence or absence of third parties is irrelevant. As a behavioural strategy, RTS is not so much concerned with partner control (forcing a partner into a cooperative interaction) but with reserving significant cooperative investment for social partners who demonstrate a willingness to do likewise. To date, there is little empirical support for RTS^17^.

Biological markets theory^10^,^11^ predicts that individuals should alter their investment in an interaction in relation to the availability of other potential partners, which may be in the form of local markets: individuals who are close at hand, i.e. bystanders. Gumert^18^ found that male long-tailed macaques (*Macaca fascicularis*) respond to local market conditions in their grooming of females, a result interpretable as a bystander effect. Unlike parcelling, however, BMT predicts only the total amount of investment as a function of market conditions, and does not predict the internal structuring of interactions. Thus while both parcelling and BMT predict a bystander effect, the division of behaviour into parcels is not predicted, or accounted for, by BMT.

Social grooming exchanges provide an excellent system within which to explore models of social cooperation^19^,^20^,^21^,^22^,^23^,^24^,^25^,^26^,^27^. Grooming provides hygienic, stress relief, and possibly thermal benefits to the recipient^28^,^29^,^30^, while the groomer has to endure possible energetic and opportunity costs^31^. While such costs may be small, also incurred by the recipient, or in some cases perhaps trivial^32^, fitness is relative and, where recipients are reproductive rivals of groomers, providing such rivals with benefits generates net costs for the groomer^33^ and even marginal gains in fitness may be important. Both parasite load and stress are driven by factors extrinsic to an individual and so will accumulate with time, creating an on-going demand for grooming that varies between individuals.

Here we test the effect of bystander presence on grooming interactions among wild chimpanzees (*Pan troglodytes*) of the M-group community of the Mahale Mountains National Park (Tanzania). We focus on adult males, as they are more gregarious than adult females and exhibit higher rates of grooming^34^,^35^. Male chimpanzees tend to engage in a broad range of mutually cooperative behaviours, such as support in agonistic interactions and sharing of food^34^,^35^ with social exchanges little influenced by kinship^36^. These males are also direct reproductive rivals in that they compete with one another for the same limited set of paternity opportunities. Chimpanzees are an ideal species with which to investigate the influence of bystanders, as the fission-fusion social system shuffles the identity and number of community members within a party^37^, so that the proximity and association between individuals can change frequently and unpredictably^38^. Occasionally, individuals of the same social group can be so far apart that defection might not be viable because the costs of searching for alternative partners would be prohibitive.

In addition, chimpanzees’ grooming bouts can take various and complex forms^17^,^24^,^25^,^27^,^39^. As with our previous work^17^,^24^,^25^, we follow Barrett *et al*.^20^ & Manson *et al*.^40^ in defining a grooming bout as an interaction between two individuals, rather than the behaviour of a single individual. Within a bout, grooming effort^24^ is typically broken up into short episodes^17^ of varying number and duration (which may correspond to the ‘parcels’ in Connor’s model), and the exchange of these can be highly dynamic. At its simplest, a bout consists of a single episode performed by one groomer, but typically a groomer will perform a sequence of episodes, interspersed by short breaks (<30 s) where the groomer is effectively resting but poised to continue grooming. In some bouts, termed ‘unidirectional’, this sequence will terminate without the recipient performing any grooming in response: the groomer may stop, or the recipient may depart. In other bouts, termed ‘bidirectional’, both individuals perform a sequence of episodes that vary in duration and may be asynchronous such that individuals alternate the roles of groomer and recipient, or overlapping such that grooming is simultaneous (‘mutual’). Sequences of grooming episodes are typically not aligned (or necessarily matched in duration), so that ‘mutual’ grooming appears to result from coincidental overlap^27^, and can end or resume purely as the result of the grooming decisions of one of the groomers. Many bouts include a combination of these structures. In only a small proportion of bouts (e.g. 3% for the Sonso community, 1994–1995^24^) do individuals start simultaneously and groom in this manner throughout.

We have reported previously our tests of both raise-the-stakes (for which we find no support^17^) and biological market theory, as applied to primate grooming^24^,^25^. Here, we test Connor’s parcelling model^12^,^13^ by exploring whether the presence of bystanders influences both the initiator’s investment and the partner’s decision to reciprocate (note that we are not concerned here with the extent to which the initiator’s partner reciprocates, but whether or not the initiator’s effort results in the receipt of grooming though a bidirectional bout).

We focus on the initial phase of a grooming bout, defined as the period during which the initiator is grooming alone before the bout either terminates as a unidirectional bout, or becomes bidirectional through the participation of the recipient. The initial phase of a grooming bout should be particularly important as the initiator is essentially gambling their investment on an unknown or uncertain outcome, whether this comes about though economic exchange or via a mechanism of relationships. Given that the groomer has yet to see any return on their investment, the initial phase should be particularly sensitive to bystander effects and initiators may abandon bouts if their current partner appears unwilling to provide the grooming in response^27^. Unlike later phases within a (bidirectional) bout, the initial phase is unaffected by previous grooming performed or received, and so any decision to abandon a bout during this period will not be influenced by the accrual of grooming benefits or a decline the rate of their accrual, while the potential effect of bystanders may diminish in later phases.

Following from Connor’s parcelling model, with more (male) bystanders we expect: less investment (i.e. less grooming effort: prediction 1), as well as fewer (prediction 2) and smaller (prediction 3) parcels (episodes of grooming), from an initiator during the initial phase of the grooming bout, and a higher probability of unidirectional bouts (prediction 4). As episodes of grooming can vary in duration, such that a given amount of grooming (grooming effort) can in principle be provided by multiple shorter episodes or fewer longer episodes, we also tested the relationships between grooming effort, number and length of episodes.

We recognise that rank differences within a dyad can influence economic decision-making if subordinates trade grooming for rank-related or rank-restricted commodities^20^,^25^,^41^ and so include social dominance rank in our analyses. ‘Grooming up the hierarchy’ is a well-known pattern in primate grooming^42^ which may be the result of lower-ranking individuals attempting to access services restricted to higher ranking individuals, such as effective agonistic support, sharing of valued food items, or tolerance^20^,^25^,^42^,^43^. However, our previous work^25^ showed that the distribution of grooming amongst the male chimpanzees of the Mahale Mountains National Park was not related to social dominance rank during the period of this study, that social tolerance was not a rank-restricted commodity, and that grooming was not exchanged for agonistic support or meat. In consequence, we do not expect to see an effect of rank difference in the tests of these predictions.

Parcel size may be contingent also on the expectation by the donor of receiving grooming from the recipient: the more likely it is that the recipient will reciprocate grooming, the bigger the donor’s parcel ought to be. Despite a growing set of findings supporting a view that primate grooming exchanges are driven by immediate economic considerations^18^,^19^,^20^,^21^,^24^,^25^,^26^,^27^,^44^,^45^,^46^,^47^, the orthodox view remains that the interchange of grooming, at least amongst catarrhine primates, functions primarily to build a platform of trust (“bonds”), which in turn facilitates fitness benefits^32^,^48^,^49^; this is why such primates groom at a level assumed to exceed the need for hygienic functions^50^,^51^. If this relationship model holds, it follows that the expectation of reciprocity in any single grooming bout should be contingent on the nature of the relationship, and thus the level of trust between individuals. If the relationship model is correct, then dyads that groom frequently and/or dyads that exchange grooming more reciprocally should be those with a greater degree of trust^21^,^22^,^48^. This conclusion is valid whether the expectation is for immediate (within-bout) or delayed (across-bout) reciprocity, and thus the amount of grooming invested by the initiator of any particular bout should be positively related to the degree of trust, the strength of the “bond” between the individuals.

Few studies have tested this prediction. Barrett *et al*.^21^ found no significant difference between frequent and infrequent grooming dyads in the duration of the first episode of grooming among chacma baboons (*Papio ursinus*). Fruteau *et al*.^26^, by contrast, did find a significant difference in first episode duration between frequent and infrequent groomers, among both mangabeys (*Cercocebus atys)* and vervet monkeys (*Chlorocebus aethiops)*, but in the opposite direction: dyads that groomed more frequently tended to give shorter first episodes than did infrequent groomers. The relationship between initial investment and the degree of reciprocity has not been investigated and we provide the first test of this among wild chimpanzees. Under the relationship model, we predict that groomer’s initial investment should be greater among frequent groomers (prediction 5) and/or strong reciprocators (prediction 6) than among infrequent groomers and weak reciprocators, respectively.

## Results

### Groomer’s initial investment

Across 774 bouts, we recorded 33 hours of grooming in the initial phase with a mean (±SD) per bout duration of 153.6 ± 179.9 s (median = 95.5 s): 139.9 ± 174 s (median = 81.5 s) during unidirectional bouts and 197.5 ± 191.6 s (median = 133.5 s) during bidirectional bouts. The initial phase of unidirectional bouts (by definition, this is equal to the total bout) consisted on average of 1.9 ± 1.6 episodes (median = 1; range = 1–13), of 72.6 ± 73.4 s (median = 51.8 s) in duration. The initial phase of bi-directional bouts consisted on average of 2.2 ± 1.7 episodes (median = 2; range = 1–9), of 96.3 ± 91.4 s (median = 74 s) in duration. Both the number of episodes and mean episode length predicted grooming effort in the initial phase (LMM: number of episodes: β ± SE = 75.99 ± 1.61, t = 47.26, p < 0.001; episode length: β ± SE = 1.387 ± 0.03, t = 41.10, p < 0.001). Number and length of episodes were not collinear (both variables, VIF = 1.00), and one did not predict the other (LMM: β ± SE = −1.821 ± 1.702, t = −1.07, p = 0.291); chimpanzees appeared to vary their grooming effort by altering either the number or length of episodes.

Only 4% of grooming bouts were interrupted; this was less likely to occur with more male bystanders (GLMM: β ± SE = −0.418 ± 0.174, t = −2.40, p = 0.02), although the number of male bystanders did not depend on the context during which the grooming interaction occurred (GLMM: β ± SE = −0.02 ± 0.062, t = −0.39, p = 0.69).

The number of male bystanders (i.e. those within 10 m) significantly predicted both groomer’s initial investment (LMM: β ± SE = −10.93 ± 5.07, t = −2.155, p = 0.03: Table 1) and number of initial episodes (GLMM: β ± SE = −0.09 ± 0.022, t = −4.26, p < 0.001: Table 2): groomers tended to invest less grooming effort through fewer episodes when there were more male bystanders, supporting predictions 1 & 2. As expected, rank distance was not a significant predictor of grooming effort (LMM: β ± SE = −0.002 ± 0.02, t = 0.13, p = 0.886: Table 1), indicating that initiators did not adjust their investment during the initial phase in relation to partner’s rank. This is consistent with our previous finding that subordinates did not give grooming in exchange for rank-related commodities (e.g. agonistic support) during the study period^25^. There was no effect of bystanders on grooming effort in bi-directional bouts after the initial phase (LMM: β ± SE = −30.71 ± 25.539, t = −1.202, p = 0.22), confirming the significance of the initial phase of grooming bouts for partner choice decisions.

In contrast with our prediction 3, there was no effect of the number of bystanders on mean episode length (LMM: β ± SE = −1.154 ± 2.219, t = −0.520, p = 0.597: Table 1), suggesting that male chimpanzees were adjusting their total investment in the initial phase of the grooming bout by changing the number of episodes, not their size. Episode length was also unaffected by the rank-distance between the initiator and recipient (LMM: β ± SE = −0.01 ± 0.009, t = 1.135, p = 0.254; Table 1). Using absolute rank (Elo-rating) of the recipient in place of rank-distance, we found that higher ranked recipients received shorter episodes in the initial phase (LMM: β ± SE = −0.027 ± 0.014, t = −1.997, p = 0.047). We found no relationship between the absolute rank of the initiator and episode length (LMM: β ± SE = −0.002 ± 0.013, t = −0.148, p = 0.881). The male chimpanzees in our sample showed a relatively flat, or structurally egalitarian, dominance hierarchy^25^; it will be interesting to see whether chimpanzees under more structurally despotic hierarchies demonstrate a more defined relationship between recipient rank and grooming episode length.

### Grooming pattern and identity of the individual who terminated the bout

#### Both the number of bystanders

**Figure 1:**
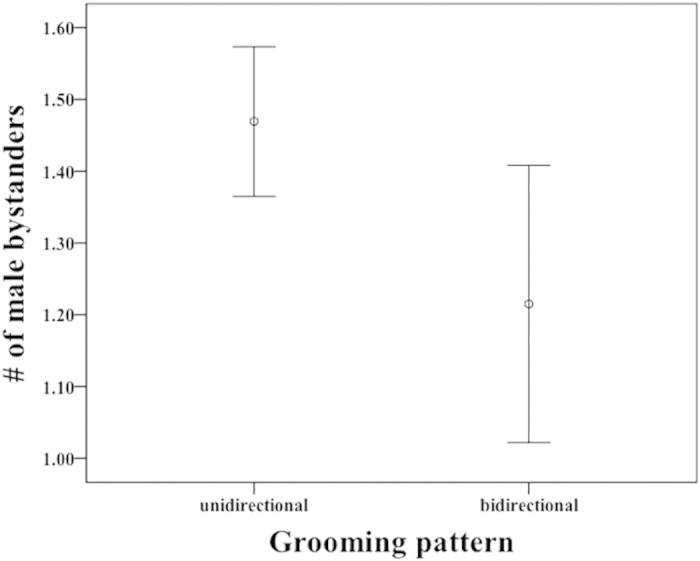
Mean number of male bystanders (within 10 m of the grooming dyad) during unidirectional and bidirectional grooming bouts. When there were more bystanders, grooming bouts tended to be unidirectional.

**Figure 2:**
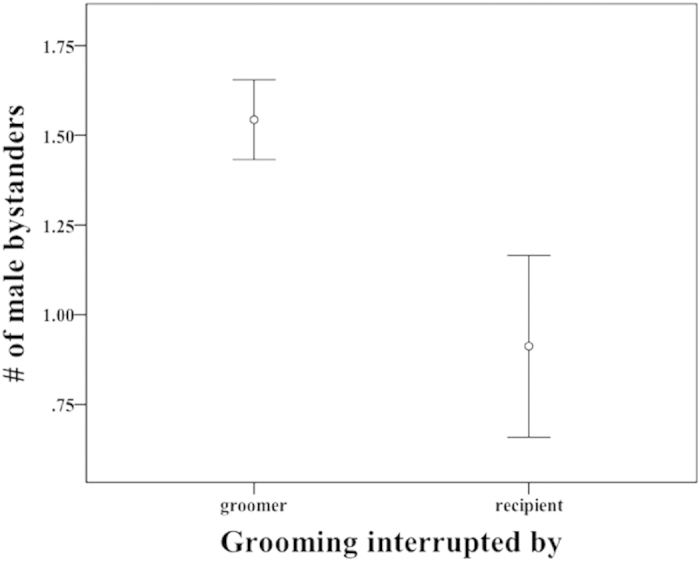
Mean number of male bystanders (within 10 m of the grooming dyad) in bouts terminated by either initiator or recipient. Initiators were more likely to terminate unidirectional grooming bouts when there were more male bystanders.

(GLMM: β ± SE = −0.158 ± 0.072, t = −2.20, p = 0.028) and rank distance (GLMM: β ± SE = 0.0008 ± 0.0003, t = 3.36, p < 0.001) predicted the occurrence of bidirectional bouts (Table 2): grooming bouts were more likely to be unidirectional when there were more males around (Fig. 1) and when rank distance between partners was larger. Since we assigned the number ‘1’ to bidirectional bouts, and positive rank distances correspond to bouts directed from dominants to subordinates, the positive relationship between grooming pattern and rank difference suggests that bouts were more likely to be bi-directional when initiated by higher-ranking males with lower-ranking partners. Furthermore, grooming was more likely to be terminated by the initiator when there were more bystanders (GLMM: β ± SE = −0.0483 ± 0.0119, t = −4.07, p < 0.001: Table 2 & Fig. 2) and during feeding contexts (GLMM: β ± SE = 0.0539 ± 0.0249, t = 2.16, p = 0.031, Table 2), a finding that suggests groomers are sensitive to opportunity costs.

### Groomer’s initial investment and trust

We classified 12 dyads as frequent groomers, while 9 dyads had a grooming reciprocity index greater than 0.8. Six dyads were both frequent groomers and strong reciprocators. We found no support for prediction 5: groomer’s initial investment was not significantly longer among frequent groomers (mean grooming ± SD: 165.26 s ± 49.21 s; median: 153.21 s) than it was among infrequent groomers (mean grooming ± SD: 159.71 s ± 84.96 s; median: 148.88 s; Mann-Whitney U test: N_frequent_ = 12, N_infrequent_ = 32, W = 209, p = 0.664). We also found no support for prediction 6: strong reciprocators did not invest significantly more in the initial phase of a bout (mean grooming ± SD: 145.64 s ± 47.62 s; median: 145 s) than weak reciprocators (mean grooming ± SD: 165.23 s ± 82.21 s; median = 152 s; N_strong_ = 9, N_weak_ = 35, W = 139, p = 0.600).

## Discussion

Our study shows a clear influence of bystanders on the initial phase of chimpanzee grooming bouts and suggests that chimpanzees use a parcelling-like strategy, at least in this phase. When more bystanders were present, we found that: (1) initiators invested less in their grooming bouts and provided both less grooming and fewer parcels; (2) bouts were less likely to be reciprocated; and (3) initiators were more likely to abandon the grooming if the recipient did not reciprocate. These results suggest that male chimpanzees make economic decisions on how much to invest in grooming interactions that are based not only on whether the partner reciprocates, but also on whether there are other potential social partners in close proximity. This is a key aspect of Connor’s parcelling model^12^,^13^. Previous studies of non-human primates have described time-matching within grooming bouts in some^20^,^24^,^40^,^43^, although not all^23^,^52^,^53^, species studied. While time-matching is consistent with parcelling, and occurs in chimpanzees^24^, our demonstration of a contingency between the initial investment in a potentially cooperative interaction, and opportunity for defection, is perhaps the strongest support yet for the parcelling model from grooming interactions of non-human primates. We add a note of caution in that our results are based on interactions of only 8 focal animals and 10 individuals in total, although this represented the entire exchange network for male chimpanzees in this social group and is comparable to other studies of this species.

Contrary to our expectations, we found evidence that dominance rank influenced episode length: initiators gave shorter episodes when they groomed high-ranking males, regardless of rank distance. Given that high-ranking individuals were more likely to terminate bouts^39^, this might suggest that lower ranking initiators anticipated a higher likelihood of non-reciprocation and limited their losses accordingly. Alternatively, short episodes may be response to risk of aggression from the higher-ranking partner: male chimpanzees have been observed to attack current grooming partners^54^, ^personal observations^, and similarly, in Japanese macaques (*Macaca fuscata*) groomers are more likely to receive aggression from recipients in post-grooming contexts than during controls^55^. Grooming requires a commitment of attention from the actor(s) and shorter episodes allow more frequent periods of vigilance to be interspersed during either the initial phase or across a complete bout. We have shown previously that chimpanzees give shorter grooming episodes when aggression rates are particularly high^17^. In this light, our finding that bi-directional grooming was more likely when bouts were initiated by high-ranking individuals might be the result of lower-ranking males being coerced into reciprocating, out of fear of receiving aggression from a more powerful individual. More generally, increased vigilance though shortening episodes may compromise grooming efficiency, result in less grooming in a bout, or extend the bout duration and so increase opportunity costs in order to maintain the amount of grooming performed or exchanged. These possibilities warrant further investigation, particularly in relation to variation between social groups in structural despotism^25^.

We found no support for predictions derived from the relationship model of primate grooming interactions. There was no influence of grooming frequency on the grooming invested in the initial phase, and we found initiators in dyads with a history of grooming reciprocity, who should under the relationship model trust their partner and expect to receive grooming in return, were no different in their initial investment to those without such a history. Thus the grooming relationship (the history of reciprocal exchanges) of a dyad appeared irrelevant to the behaviour of an individual initiating a grooming bout, and we have no evidence that individuals who had received high levels of reciprocity in the past from their current partner trusted that they would do so again. This finding calls into question the concept that grooming builds trust between individuals. Our findings echo the results of Machanda *et al*.^27^, who also found no support for predictions derived from the relationship model in their study of simultaneous, or mutual, grooming in chimpanzees, as well as our recent finding that male grooming of cycling (‘oestrus’) females could not be explained by the relationship model^56^. In light of these results, and the growing body of work supporting a markets-based model of economic exchange^18^,^19^,^20^,^21^,^24^,^25^,^26^,^27^,^44^,^45^,^46^,^47^, a critical re-evaluation of the appropriateness of the relationship (‘bonding’) model as an explanation for grooming in chimpanzees, as well as other catarrhine primates, seems overdue^33^. It may even be the case that immediate benefits drive most if not all social interactions: Gilby^57^ found this to be the case for meat sharing, with individuals in possession of carcasses paying harassing ‘beggars’ to leave in order to increase their own intake rates rather than sharing food with frequent grooming partners (surprising only under the assumption that grooming builds social bonds).

We have presented evidence elsewhere as to the importance of market forces on the grooming decisions of male chimpanzees^25^. From a market forces perspective, bystander effects can be seen as the product of partner choice strategies responding to local economic conditions, implying that individuals make choices within local markets – their immediate social environments^18^ – rather than a broader market presented by their global social environment (the social group, or population, depending on social dynamics of particular species). As a consequence of search and opportunity costs, local markets become essentially isolated from one another. However, biological market theory is a model to account for the evolution of partner choice strategies^10^,^11^,^41^ and, as such, individuals embodying those strategies should be equipped to respond to average market conditions (levels of supply and demand) across their global market, as well as evaluating local conditions. Thus a biological markets model should predict that partner choice decisions depend on local market conditions – bystanders – weighted by expected average returns from selecting instead from the supply in the global market. While local markets might be isolated from one another, they should be regarded as nested within a global market. Such nesting and the consequent bystander effects potentially complicate tests of biological market models. In species such as chimpanzees where a social group (the global market place for an individual) is fragmented into discrete sub-groups (parties), or the macaques studied by Gumert^18^, it may be necessary to consider both local and global market conditions, the later devalued by average search and opportunity costs, when determining the level of supply or demand to which an animal may be responding. That said, structuring of social groups into discrete sub-groups also provides opportunities to test between different market conditions within a single social group, should local conditions vary sufficiently that individuals respond to these differences when choosing social partners. More generally, existence of bystander effects and local markets suggest that search costs should be given more explicit consideration in testing biological market models across taxa.

Here, we focused on the initial phase of grooming bouts and showed that this is particularly important for partner-choice decisions, with the presence of bystanders having no effect on later phases of bidirectional bouts. If we consider bystanders to represent a local market of alternative suppliers of grooming, then the question is why we need to consider parcelling at all as an explanation ? The obvious answer is that chimpanzees deliver their grooming effort in discrete episodes within bouts – they parcel their grooming – an effect that is not predicted (although not precluded) by biological market theory. Furthermore, individuals continue to parcel their grooming throughout bidirectional bouts^17^, but without an effect of bystanders (or local markets). Parcelling provides an additional layer of explanation to that offered by biological market theory and, given the complexity of these grooming interactions, we feel that parcelling should be seen as a strategy available to chimpanzees rather than a model that can fully account for the evolutionary dynamics of cooperative grooming exchanges.

Overall, our study shows that male chimpanzees modify the amount of grooming investment in the initial phase of the bout in relation to the number of male bystanders: when there are more males in close proximity, initiators tend to give less grooming, with fewer grooming episodes, and are more likely to interrupt a bout in the absence of immediate reciprocation, making unidirectional bouts more likely to occur. Our results show that the number of bystanders present during a grooming interactions can affect parcel size, at least in terms of the amount of grooming invested, as predicted by Connor’s model and highlight the importance of considering the presence of other individuals during cooperative interactions, and the distinction between local and global markets, as a way towards a better understanding of the evolved behavioural strategies that generate cooperative interactions.

## Methods

### Study site and subjects

We studied the grooming behaviour of adult male chimpanzees of M-group community from the Mahale Mountains National Park, Tanzania (06°15′S; 29°55′E), between February and November 2011. This community has been continuously studied for over 30 years^35^; as a result our study subjects were fully habituated to human observation, and grooming interactions could be recorded in detail at close range. The community consisted of 60 individuals, including ten adult males (>16 years old) and 23 adult females (>14 years old)^17^. This research was carried out in accordance with the approved guidelines for the ethical treatment of primates, was approved by the Ethics Committee of University of Kent and adhered to the legal requirements of Tanzania and the UK.

### Data collection and analysis

We collected data through long-day focal follows on eight adult males. Data on grooming and agonistic interactions were collected using all-occurrence sampling^58^ within focal parties (i.e. all occurrences of these interactions that occurred in a party that contained a nominal focal animal, where party is defined as a subgroup produced by the fluid fission-fusion social system). Thus we sampled the grooming interactions of all 10 adult males. Each day the focal animal with the fewest hours of observation was selected in order to balance the number of hours of observation across adult males (logistic constraints meant that we did not select two males as focal animals), and this individual was followed when parties fissioned. Parties were followed for as long as possible from first encounter until nesting. If contact with chimpanzees was lost due to terrain and/or chimpanzee movement patterns, we searched for and observed the next party encountered that contained one of the predetermined focal animals. We recorded data through audio narration, or by pen and paper. We conducted 141 focal follows for a total of 800.9 h of observation.

We defined grooming as the visual examination, searching and manipulation of the skin and hair with one or both hands, with the occasional use of the lower lip to part the hair. When a male initiated a grooming bout, we recorded: (1) the identity of the individual who approached the partner either to give or receive grooming, together with the time of the approach; (2) the identity of other males within 10 m (whom we labelled bystanders); (3) the changes in the identity of groomer and recipient, including the initiator; (4) the end time of the grooming bout, and the identity of the individual who terminated the bout. A grooming bout was considered to have ended when neither individual groomed for at least 30s^24^,^25^. We recorded the context in which grooming occurred, on the basis of the activity of the grooming dyad prior to the beginning of the bout, as well as the activity of the majority of the male bystanders. We classified three types of contexts: feeding, resting and travelling. Finally, we noted whether the grooming bout was interrupted by aggression either from third parties or performed by one of the participants in the grooming bout.

We examined grooming bouts as pairwise (dyadic) interactions. Bouts were classified as either bidirectional when both individuals contributed some grooming^20^, whether simultaneously or by taking turns, or unidirectional when only one individual groomed. A grooming episode was defined as an uninterrupted period of grooming given by one individual, ending when neither of groomer’s hands was in contact with partner’s body^17^. A groomer’s initial investment was defined as the amount (duration) of grooming given by an individual at the beginning of a bout before either the bout was terminated (resulting in an unidirectional bout) or the receipt of grooming from the partner (resulting in bidirectional bout). For the purposes of data analysis, we excluded bouts that started prior to the observation and those whose pattern could not be accurately described due to poor visibility. Similarly, bidirectional bouts in which a clear initiator could not be identified (e.g. if partners started grooming each other simultaneously: n = 66 bouts) were discarded. This gave us a total of 774 grooming bouts: 588 unidirectional and 186 bidirectional.

To determine social dominance rank we used the outcome of agonistic interactions to calculate dominance rank order using Elo-ratings^25^,^59^ implemented in R 3.2.1. We defined agonistic interactions as those instances in which a male attacked another group member either through physical contact (e.g. slap, bite) or charging displays and/or chasing^17^,^25^. We determined rank difference by subtracting the Elo-rating of the recipient from the Elo-rating of the groomer. Since high Elo-rating values correspond to high ranks^59^, negative rank differences correspond to grooming interactions directed from subordinates to dominants.

We tested whether groomer’s investment in the initial phase was predicted by the number and mean length of episodes in this phase, using the *lmer* function in the package ‘lme4’ (in R 3.1.2) to perform a Linear Mixed Model (LMM) analysis. We set grooming effort (i.e. duration) as the dependent variable, while the number and mean duration of initial episodes were entered as independent fixed factors. We ran a similar model to assess whether mean episode length predicted the number of episodes. We used the *drop1* function in R to compute a likelihood ratio test and thereby assess whether the independent factor(s) exerted a significant effect on the dependent variable.

To assess whether the number of male bystanders significantly affected the likelihood that a grooming bout was interrupted, and depended on the context of the grooming interaction, we ran two different Generalized Linear Mixed Model (GLMM) analyses, using the *glmer* function in the package ‘lme4’. In one model we included information on whether or not the bout was interrupted as binomial dependent variable, with the number of bystanders set as independent factor. In a separate model, we considered the number of male bystanders as a dependent variable entered as count data with Poisson distribution, while the context was included as an independent factor.

To test whether groomers’ initial investment (prediction 1), and the mean length of grooming episodes (prediction 3) were affected by the number of male bystanders and rank difference between partners, we ran two Linear Mixed Model analyses with the *lmer* command and the *drop1* function in R 3.1.2. The initiator’s initial investment and the mean length of episodes were entered as continuous dependent variables in separate models, while in each model the number of male bystanders, rank difference between partners, the context during which the grooming interaction occurred and a binary variable describing whether the bout was interrupted were set as fixed factors. To determine whether the initial phase was distinct from the remainder of bi-directional bouts in its sensitivity to bystander effects (one of our initial assumptions), we investigated whether the remaining investment provided by an initiator was influenced by the number of bystanders and/or the rank distance between partners. To this end, we used an LMM analysis with the total grooming effort in each bout, minus that in the respective initial phase, as the dependent variable and with the number of bystanders and rank distance between partners as fixed factors.

We used a Generalized Linear Mixed Model with negative binomial distribution to assess the effect of bystander and dominance rank on the number of grooming episodes given by initiators (prediction 2) in the initial phase of the bout, using the *glmmadmb* function in R 3.1.2. In this model, the number of episodes was entered as count data, with the number of male bystanders, rank difference, context and whether the bout was interrupted included as fixed factors, as above. We used two Generalized Linear Mixed Models with binomial distributions (logistic GLMM) to test (a) whether reciprocation was less likely with more bystanders (i.e. bouts were more likely to be unidirectional, prediction 4), and (b) whether the initiator or the recipient were more likely to terminate a unidirectional bout in the presence of bystanders, again using the *glmmadmb* function. In the first of these models, we set grooming pattern (unidirectional vs. bidirectional) as the dependent variable; in the second, the identity of the individual who terminated the bout (initiator vs. recipient; only for unidirectional bouts). The number of bystanders, rank difference, context and whether the bout was interrupted were entered as fixed factors in both models.

In all these models (LMM and GLMM), the identity of the dyad and the day on which each bouts occurred (as a category variable, such that all bouts on the same day were given the same value) were included as random factors (intercepts) with nested structure (within-day bouts nested within dyads) in order to control for the different contribution that each dyad gave to the data set, and for the non-independency of the grooming bouts that were exchanged within a day.

To test predictions 5 and 6, we categorised dyads on the basis of the frequency of grooming exchanged and the strength of grooming reciprocity. To identify the dyads that exchanged grooming time more frequently, we calculated the time spent grooming (grooming effort) for each dyad as a proportion of the total observation time for both members of that dyad. We ranked dyads on this proportion from the highest to lowest and, following Koski *et al*.^60^, defined dyads in the top quartile as frequent groomers whilst the other dyads were considered infrequent groomers. To determine the degree of grooming reciprocity for each dyad, we calculated its reciprocity index RI^24^,^25^:


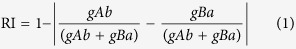


in which *g*Ab is the grooming that individual A directed towards B, *g*Ba is the grooming that B directed towards A and *g*Ab + *g*Ba is the total grooming exchanged between A and B. This index can range between 0 (no reciprocity) and 1 (complete reciprocity). Dyads with an RI equal or greater than 0.8 were defined as strongly reciprocating^24^.

We used Mann-Whitney U tests to compare the groomer’s initial investment across dyads. As initial grooming investments in different bouts are potentially not independent when they occur within the same dyad, we calculated mean groomer’s initial investment for each dyad and compared these values between dyads that were frequent and infrequent groomers, and between the strongly reciprocating and other dyads.

## Additional Information

**How to cite this article**: Kaburu, S. S. K. and Newton-Fisher, N. E. Bystanders, parcelling, and an absence of trust in the grooming interactions of wild male chimpanzees. *Sci. Rep.* **6**, 20634; doi: 10.1038/srep20634 (2016).

## Figures and Tables

**Table 1:** Mixed model analyses showing the effect of the number of male bystanders and rank difference on the duration of groomer’s initial investment and the mean episode length.

Analysis	Dependent	Predictor	β	SE	t	p
LMM	Initial investment	Intercept	200.014	19.715	10.145	—
		Rank distance	0.002	0.020	0.130	0.886
		**# of bystanders**	**−10.93**	**5.070**	**−2.155**	**0.03**
		Context	−21.860	13.386	−1.633	0.103
		Interrupted	−40.852	31.237	−1.308	0.188
LMM	Mean episode length	Intercept	90.402	8.55	10.571	—
		Rank distance	0.01	0.009	1.135	0.254
		# of bystanders	−1.154	2.219	−0.520	0.597
		Context	−7.385	5.861	−1.260	0.207
		Interrupted	−16.740	13.698	−1.222	0.219

Significant results are presented in bold.

**Table 2:** Generalized Mixed Model analysis showing the effect of rank distance and number of bystanders on grooming pattern (unidirectional vs. bidirectional) and the identity of the individual who terminated the grooming bout (initiator vs. recipient).

Analysis	Dependent	Predictor	β	SE	z	p
Negative binomial GLMM	Number of episodes	Intercept	0.907	0.0785	11.56	<0.001
		Rank distance	−1.08 × 10^−4^	8.46 × 10^−5^	−1.28	0.20
		**# of bystanders**	**−0.09**	**0.022**	**−4.26**	**<0.001**
		Context	−0.08	0.057	−1.39	0.16
		Interrupted	−0.097	0.136	−0.72	0.47
GLMM	Grooming pattern	Intercept	−1.126	0.267	−4.21	<0.001
		**Rank distance**	**0.0008**	**0.0003**	**3.36**	**<0.001**
		**# of bystanders**	**−0.158**	**0.072**	**−2.20**	**0.028**
		Context	0.08	0.183	0.44	0.661
		Interrupted	0.314	0.400	0.79	0.432
Binomial GLMM	Who terminates bout	Intercept	−1.95	0.035	−5.63	<0.001
		Rank distance	**−**6.07 × 10−5	3.91 × 10^−4^	−0.16	0.876
		**# of bystanders**	**−0.0483**	**0.0119**	**−4.07**	**<0.001**
		**Context**	**0.0539**	**0.0249**	**2.16**	**0.031**
		Interrupted	−0.007	0.0575	−0.01	0.990
